# Combined proximal tubulopathy, crystal-storing histiocytosis, and cast nephropathy in a patient with light chain multiple myeloma

**DOI:** 10.1186/s12882-017-0584-8

**Published:** 2017-05-25

**Authors:** Chung-Kuan Wu, An-Hang Yang, Hung-Chih Lai, Bing-Shi Lin

**Affiliations:** 1 Division of Nephrology, Department of Internal Medicine, Shin-Kong Wu Ho-Su Memorial Hospital, 95,Wen Chang Rd., Shih Lin District, Taipei, 11101 Taiwan; 2 Institute of Clinical Medicine, National Yang-Ming University, Taipei, Taiwan; 3 Department of Pathology and Laboratory Medicine, Taipei Veterans General Hospital, Taipei, Taiwan; 4 Division of Hematology and Oncology, Department of Internal Medicine, Shin-Kong Wu Ho-Su Memorial Hospital, Taipei, Taiwan; 5 School of Medicine, National Yang-Ming University, Taipei, Taiwan

**Keywords:** Multiple myeloma, Light chain proximal tubulopathy, Crystal-storing histiocytosis, Myeloma cast nephropathy

## Abstract

**Background:**

The diagnosis of myeloma, a plasma dyscrasia, often results from the workup of unexplained renal disease. Persistent renal failure in myeloma is commonly caused by tubular nephropathy due to circulating immunoglobulins and free light chains. Myeloma cast nephropathy is characterized by crystalline precipitates of monoclonal light chains within distal tubules. Immunoglobulin crystallization rarely occurs intracellularly, within proximal tubular cells (light chain proximal tubulopathy) and interstitial histiocytes (crystal-storing histiocytosis). We present a case report of a rare simultaneous occurrence of light chain proximal tubulopathy, crystal-storing histiocytosis, and myeloma cast nephropathy in a patient with κ light chain multiple myeloma.

**Case presentation:**

A 48-years-old man presented with uremia and anemia. Laboratory examination revealed low levels of serum IgG, IgA, and IgM. Serum and urine immunofixation electrophoresis showed a free κ monoclonal band. Bone marrow aspiration and biopsy revealed hypercellularity with marked plasmacytosis. Light microscopy revealed eosinophilic cuboid- and rhomboid-shaped crystals in the cytoplasm of proximal tubular epithelial cells, diffuse large mononuclear and multinuclear cells in the interstitium, and obstructed distal tubules with cast and giant cell reaction. Immunohistochemical examination indicated intense staining for κ light chains within casts, histiocytes, and tubular epithelial cells. Electron microscopy revealed electro-dense cuboid-, rhomboid-, or needle-shaped crystalline inclusions in proximal tubular epithelial cells and interstitial histiocytes. According to these results, we confirmed that this patient with myeloma exhibited simultaneous light chain proximal tubulopathy, crystal-storing histiocytosis, and myeloma cast nephropathy, which were attributed to monoclonal κ light chains. In addition to dialysis, the patient received induction chemotherapy with a combination of bortezomib, cyclophosphamide, and dexamethasone, followed by maintenance therapy with thalidomide. However, the patient did not regain renal function even when less than 5% plasma cells were detected in the bone marrow.

**Conclusion:**

To the best of our knowledge, this is the first report of simultaneous light chain proximal tubulopathy, crystal-storing histiocytosis, and myeloma cast nephropathy in κ light chain multiple myeloma.

## Background

Multiple myeloma (MM) is a malignant neoplasm arising from clonal proliferation of plasma cells in the bone marrow; MM can cause renal dysfunction through various mechanisms, including paraprotein- or nonparaprotein-associated renal complications [1]. Persistent renal failure in MM commonly results from tubular nephropathy due to circulating paraproteins of various types secreted by plasma cell clones, most commonly immunoglobulins(Igs) and free light chains (LCs) [2]. Myeloma cast nephropathy (MCN), the most common Ig-related crystalline nephropathy, is characterized by crystalline precipitates of monoclonal LC (either κ or λ) within distal tubules [1]. On rare occasions, Ig crystallization occurs intracellularly within proximal tubular cells (LC proximal tubulopathy [LCPT]) [3, 4] and interstitial histiocytes (crystal-storing histiocytosis [CSH]) [5, 6]. In LCPT and CSH, these reabsorbed LCs are typically of κ type and possess innate physicochemical properties that resist proteolysis and promote self-aggregation and crystal formation [7–9]. Recently, the pathologic spectrum of LCPT has been expanded to include noncrystalline morphology [10–12].

Here, we report a rare case of myeloma with combined LCPT, CSH, and MCN attributable to free κ LC that presented clinically as uremia and anemia.

## Case presentation

A 48-years-old man with urolithiasis history experienced dizziness, anorexia, and shortness of breath for 2 weeks. During this period, he also exhibited nausea and vomiting. Physical examination revealed a pale and ill-looking patient with blood pressure of 135/71 mmHg, blood temperature of 36.8 °C, and a pulse rate of 86 beats/min. The hemogram revealed hematocrit of 16.3%, a leukocyte count of 1.24 × 10^4^/μL, and a platelet count of 9.8 × 10^4^/μL. The biochemical assay results were as follows: blood urea nitrogen, 94 mg/dL; serum creatinine, 12.6 mg/dL; uric acid, 11.5 mg/dL; sodium, 135 mmol/L; potassium, 4.2 mmol/L; ionized calcium, 5.72 mg/dL; phosphate, 6.1 mg/dL; serum iron, 110 μg/dL; total iron binding capacity, 305 μg/dL; and ferritin, 647 ng/mL. Urinalysis results were 1+ for occult blood and 1+ for protein. The urine microalbumin-to-creatinine ratio and urine total protein-to-creatinine ratio were 36.25 mg/g and 3.53, respectively.

The patient was initially treated with hemodialysis and blood transfusion. Examination of serum Ig revealed low IgG, IgA and IgM levels, which were 302 mg/dL (751–1560 mg/dL), 10 mg/dL (82–453 mg/dL), and 9.3 mg/dL (46–304 mg/dL), respectively. Examination of serum complement (C) revealed normal C3 and high C4 levels, which were 135 mg/dL and 53.6 mg/dL, respectively. Serum and urine immunofixation electrophoresis showed a free κ monoclonal band. Beta-2 microglobulin was higher than 5 × 10^4^ ng/mL (609–2366 ng/mL). Long bone and skull radiography revealed osteolytic foci in the inferior ramus of the bilateral pubic bones and no punched out lucencies in the skull. Bone marrow aspiration revealed a monotonous pattern with marked plasmacytosis and mononuclear cell distribution of 57%. Bone marrow biopsy revealed hypercellularity with diffuse infiltration of plasmacytoid cells, and more than 50% of the cells exhibited positive immunohistochemical staining for CD138 and κ chains. Additional laboratory data revealed negative serum antibodies against HIV, hepatitis B and C, syphilis, and negative antinuclear antibody.

**Fig. 1:**
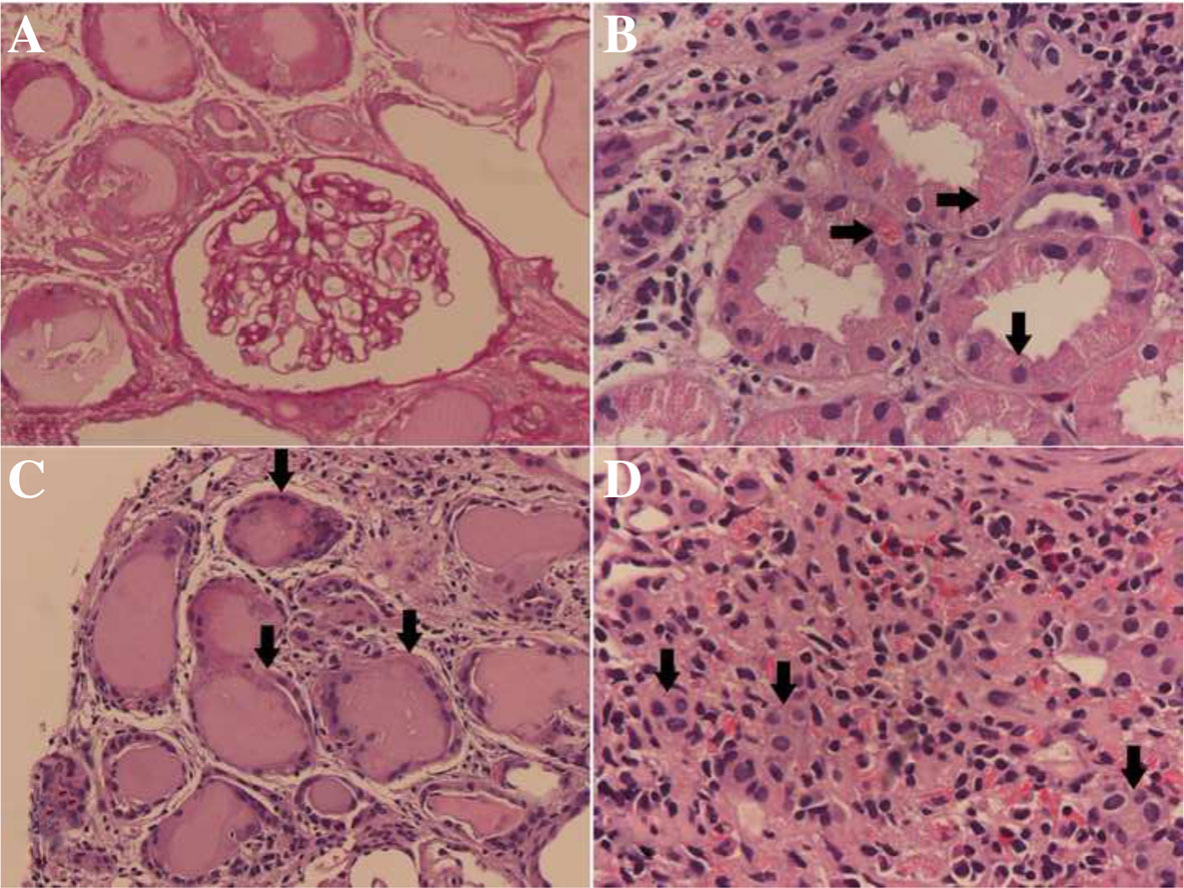
Light microscopic features of proximal tubulopathy, histiocytosis, and cast nephropathy. **a** Segmental sclerosis of glomerulus (Periodic acid-Schiff). **b** Eosinophilic cuboid and rhomboid crystals (*arrows*) in the cytoplasm of proximal tubular epithelial cells. **c** Giant cell reaction around the casts and obstructed distal tubule with fractured dense cast (*arrows*). **d** Many mononuclear and multinuclear cells (*arrows*) in the renal interstitium (hematoxylin and eosin)

**Fig. 2:**
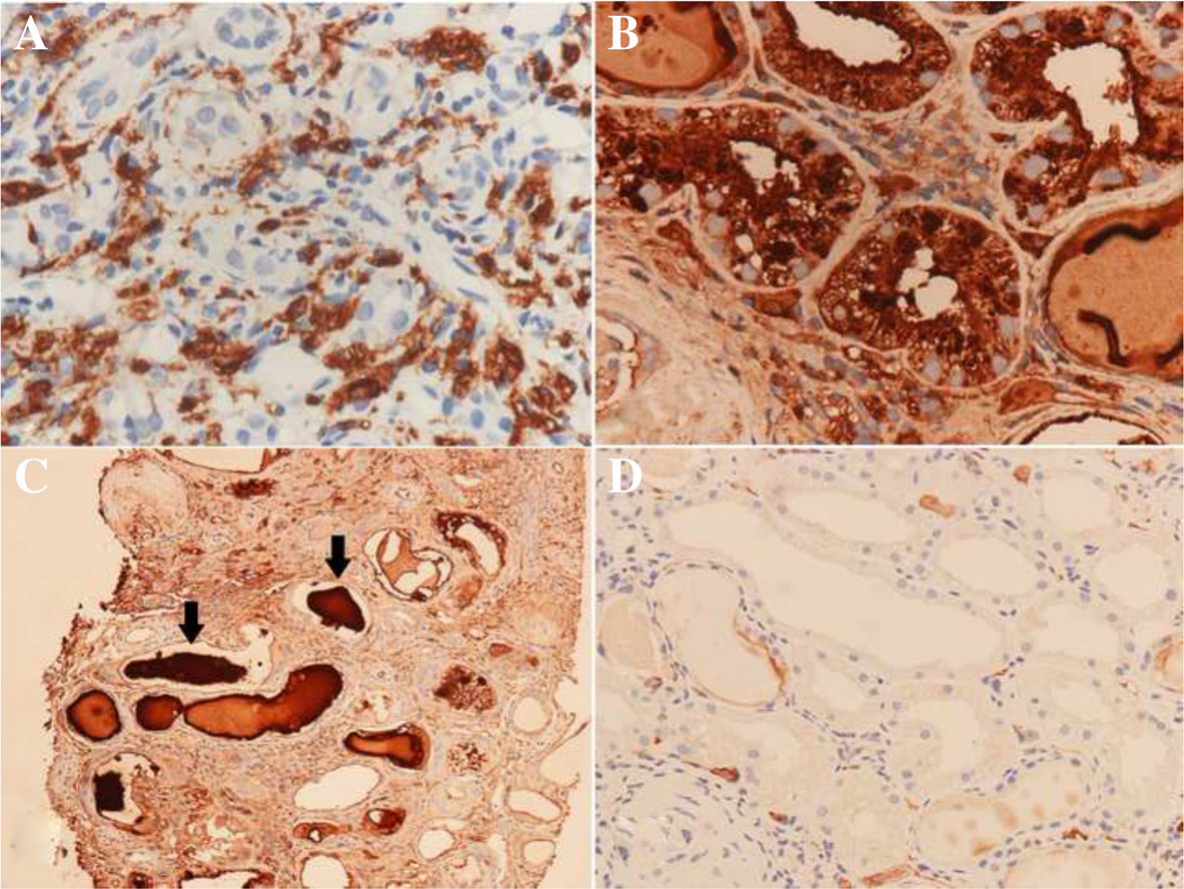
Immunohistochemical staining for renal involvement of monoclonal *k* light chain gammopathy. **a** CD68 immunostaining showed reactivity in interstitial mononuclear and multinucleated cells. **b** κ light chain staining in tubular epithelial cells. **c** κ light chain staining in casts (*arrows*). **d** λ light chains were not detected

**Fig. 3:**
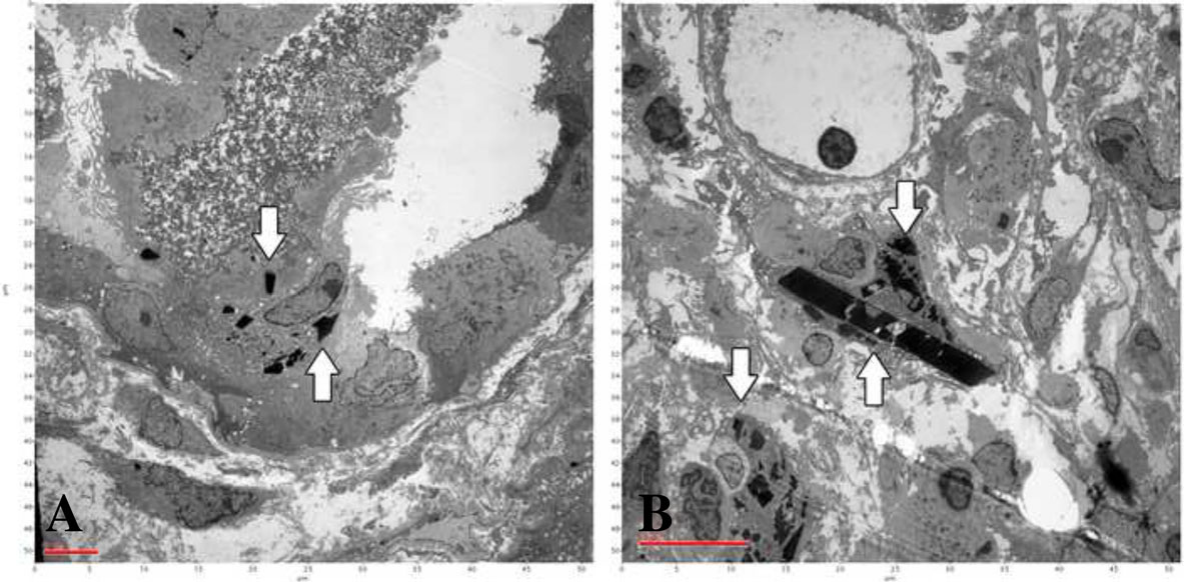
Electron microscopy showing crystalline inclusions in proximal tubular epithelial cells and histiocytes. **a** Cuboid- and rhomboid-shaped crystalline inclusions (*arrows*) in proximal tubular epithelial cells. **b** Needle- and rhomboid-shaped crystalline inclusions (*arrows*) in histiocytes

Renal sonography showed a normal size for both kidneys. Examination of all material from percutaneous ultrasound-guided renal biopsy revealed a total of 30 glomeruli per level section, of which 12 were globally sclerosed and 4 were segmentally sclerosed (all of “no otherwise specified” variant) (Fig. 1a). Renal tubules demonstrated more than 50% tubular atrophy. Eosinophilic crystals in the cytoplasm of proximal tubular epithelial cells (Fig. 1b) and obstructed tubules with fractured dense cast and giant cell reaction (Fig. 1c) were also observed. More than 50% of the renal interstitium exhibited interstitial fibrosis and inflammatory change. Diffuse mononuclear cells, plasma cells, and histiocytes (Figs. 1d, 2a) were observed in the renal interstitium. Immunohistochemical studies of κ and λ LCs revealed intense staining for κ LCs within casts, histiocytes, and tubular epithelial cells (Fig. 2b, c) but negative staining for λ LCs (Fig. 2d). Electron microscopy showed irregular effacement of podocyte foot process and electro-dense cuboid- and rhomboid-shaped crystals in proximal tubular epithelial cells (Fig. 3a) and electro-dense rhomboid- or needle-shaped crystalline inclusions in interstitial histiocytes (Fig. 3b).

The patient was treated using induction chemotherapy with a combination of bortezomib, cyclophosphamide, and dexamethasone (VAD) for eight cycles. After induction therapy, the beta-2 microglobulin level decreased to 9212 ng/mL, and serum IgG increased to 950 mg/dL. Bone marrow biopsy revealed less than 5% plasma cells. Subsequently, the patient received maintenance therapy with thalidomide. Nevertheless, his renal function did not recover, and he continues to undergo hemodialysis.

## Discussion

MM, a malignant plasma cell disorder, is defined by the presence of a serum monoclonal spike (M-spike) of more than 3 g/dL or more than 10% clonal plasma cells in the bone marrow and at least one myeloma-defining event, such as hypercalcemia, renal impairment, anemia, or bone lesions [13]. The kidney is a major target organ, and renal impairment is frequently the first manifestation of the disease. Renal impairment occurs in up to 40% of patients and 10–20% will require dialysis [14]. In our patient, more than 50% of the cells exhibited positive staining for CD138 and κ chain in the bone marrow. Moreover, the patient presented with hypercalcemia, renal failure, anemia, and pubic bone lesions. Hence, a diagnosis of MM was made.

Monoclonal Ig LCs are the major causes of renal complications in MM. Renal disease in most patients with myeloma is caused by MCN [15]. Most cases of MCN occur in patients with serum free LCs (FLCs) above 100 mg/dL, and FLCs less than 70 mg/dL are rarely observed [16, 17]. These high FLC concentrations overwhelm the reabsorption capacity of proximal tubules; thus, FLCs pass into the loop of Henle, where they bind to the Tamm-Horsfall protein and subsequently aggregate to form casts [18]. Histologically, MCN is characterized by the presence of intratubular LC casts in the distal tubules and collecting ducts. The casts often have a “hard” and “fractured” appearance. Giant cell reaction is commonly observed around the casts, because mononuclear cells are recruited in an attempt to remove these casts. Pathologically, our patient exhibited typical hard and fractured myeloma casts in the distal tubules with giant cell reaction.

Myeloma-associated renal Fanconi syndrome is a rare disorder characterized by proximal tubular dysfunction due to reabsorption of monoclonal Ig LCs, nearly invariably of the κ type [19]. These LCs possess innate physicochemical properties that resist proteolysis and promote self-aggregation and crystal formation [3, 8, 20]. The pathologic spectrum of LCPT has been expanded to include noncrystalline morphology. Patients with noncrystalline LCPT may exhibit droplets, granules, or vacuoles in the cytoplasm of proximal tubular cells [21]. Clinically, our patient was diagnosed with κ LC MM. Pathologically, proximal tubular cells were distended with intracytoplasmic cuboidal or rhomboidal inclusions with the focal loss of brush border.

CSH, an uncommon phenomenon in disorders associated with the expression of monoclonal Ig, is defined as the accumulation of histiocytes in the bone marrow and the presence of large histiocytes containing numerous Ig crystals at extramedullary sites [9]. It is presumed to be an intralysosomal accumulation of secreted paraproteins or Ig, which aggregate in crystals. In CSH, κ LCs of Ig are almost exclusively involved without a consistent association of a particular heavy chain [9]. The bone marrow is the most common site of histiocytes accumulation, whereas histiocytes containing crystals in the kidney have rarely been reported. Patients with CSH-associated renal disorder exhibit tubule-interstitial lesions and proximal tubular lesions [22–24]. Our patient predominately exhibited interstitial lesions with infiltration of large mononuclear cells and histiocytes containing rhomboid- and needle-shaped crystalline inclusions accompanied by interstitial fibrosis and tubular atrophy.

The main goal of therapy in MM is to reduce light chain production through chemotherapy alone or followed by autologous stem cell transplant (ASCT). For newly diagnosed MM, the preferred combinations for induction therapy include bortezomib, lenalidomide, and dexamethasone (VRD); carfilzomib, lenalidomide, and dexamethasone (KRD); and bortezomib, cyclophosphamide, and dexamethasone (VCD). The drug choice is determined by the risk classification of MM and the patient’s clinical status [25]. Because LCPT and CSH are relatively rare forms of renal disease in MM according to medical literature, no preferred chemotherapy regimen has been mentioned, and ASCT results in stable or improved renal function in patients with crystalline LCPT [21]. Our patient received induction therapy with bortezomib, cyclophosphamide, and dexamethasone (VCD) for eight cycles because of his poor renal function.

Patients with cast nephropathy who present with advanced renal failure are most likely to exhibit irreversible diseases, with >80% requiring dialysis at presentation and only 15% regaining renal function [26]. The prognosis of patients on dialysis depends on the response to chemotherapy, with responders surviving 37 months compared with 12 months for nonresponders [27]. Prognosis and optimal therapy for LCPT remain largely unknown. Although the majority of patients exhibit indolent kidney dysfunction, more precipitous development of acute renal failure infrequently occurs. Median renal survival from the time of renal biopsy is shorter for noncrystalline LCPT (64 ± 17.8 months) than for crystalline LCPT (135 ± 5.5 months) [21]. In the absence of renal failure, many MM patients with CSH exhibit higher survival rates after diagnosis than do patients with MM alone [9]. However, renal involvement in CSH is uncommon; the prognosis of CSH with renal involvement remains unclear. Our patient exhibited advanced renal failure accompanied by monoclonal LC-related renal disorders, including MCN, crystalline proximal tubulopathy, and CSH. After induction chemotherapy, the patient’s renal function still did not recover even when his beta-2 microglobulin level and serum IgG improved and when less than 5% plasma cells were detected in the bone marrow.

## Conclusion

In summary, we report a rare simultaneous occurrence of LCPT, CSH, and MCN in a patient with MM. This patient exhibited a wide spectrum of renal lesions caused by monoclonal κ LC proliferations, and renal prognosis was as poor as that for MCN.

## Abbreviations

ASCT: Autologous stem cell transplant
CSH: Crystal-storing histiocytosis
FLC: Free light chain
Ig: Immunoglobulin
LC: Light chain
LCPT: Light chain proximal tubulopathy
MCN: Myeloma cast nephropathy
MM: Multiple myeloma
